# Long-term Satellite NDVI Data Sets: Evaluating Their Ability to Detect Ecosystem Functional Changes in South America

**DOI:** 10.3390/s8095397

**Published:** 2008-09-03

**Authors:** Germán Baldi, Marcelo D. Nosetto, Roxana Aragón, Fernando Aversa, José M. Paruelo, Esteban G. Jobbágy

**Affiliations:** 1 Grupo de Estudios Ambientales - IMASL, Universidad Nacional de San Luis & CONICET. Ejército de los Andes 950, D5700HHW San Luis, Argentina; 2 Cátedra de Métodos Cuantitativos Aplicados, Facultad de Agronomía, Universidad de Buenos Aires, Av. San Martín 4453, C1417DSE Buenos Aires, Argentina; 3 Universidad Nacional de San Luis. Ejército de los Andes 950, D5700HHW San Luis, Argentina; 4 Laboratorio de Análisis Regional y Teledetección – IFEVA & Facultad de Agronomía, Universidad de Buenos Aires & CONICET. Av. San Martín 4453, C1417DSE Buenos Aires, Argentina

**Keywords:** Ecosystems, time series analysis, NOAA-AVHRR, NDVI, PAL, GIMMS, FASIR, South America

## Abstract

In the last decades, South American ecosystems underwent important functional modifications due to climate alterations and direct human intervention on land use and land cover. Among remotely sensed data sets, NOAA-AVHRR “Normalized Difference Vegetation Index” (NDVI) represents one of the most powerful tools to evaluate these changes thanks to their extended temporal coverage. In this paper we explored the possibilities and limitations of three commonly used NOAA-AVHRR NDVI series (PAL, GIMMS and FASIR) to detect ecosystem functional changes in the South American continent. We performed pixel-based linear regressions for four NDVI variables (average annual, maximum annual, minimum annual and intra-annual coefficient of variation) for the 1982-1999 period and (1) analyzed the convergences and divergences of significant multi-annual trends identified across all series, (2) explored the degree of aggregation of the trends using the O-ring statistic, and (3) evaluated observed trends using independent information on ecosystem functional changes in five focal regions. Several differences arose in terms of the patterns of change (the sign, localization and total number of pixels with changes). FASIR presented the highest proportion of changing pixels (32.7%) and GIMMS the lowest (16.2%). PAL and FASIR data sets showed the highest agreement, with a convergence of detected trends on 71.2% of the pixels. Even though positive and negative changes showed substantial spatial aggregation, important differences in the scale of aggregation emerged among the series, with GIMMS showing the smaller scale (≤11 pixels). The independent evaluations suggest higher accuracy in the detection of ecosystem changes among PAL and FASIR series than with GIMMS, as they detected trends that match expected shifts. In fact, this last series eliminated most of the long term patterns over the continent. For example, in the “Eastern Paraguay” and “Uruguay River margins” focal regions, the extensive changes due to land use and land cover change expansion were detected by PAL and FASIR, but completely ignored by GIMMS. Although the technical explanation of the differences remains unclear and needs further exploration, we found that the evaluation of this type of remote sensing tools should not only be focused at the level of assumptions (i.e. physical or mathematical aspects of image processing), but also at the level of results (i.e. contrasting observed patterns with independent proofs of change). We finally present the online collaborative initiative “Land ecosystem change utility for South America”, which facilitates this type of evaluations and helps to identify the most important functional changes of the continent.

## 1. Introduction

### 1.1. The detection and quantification of ecosystems changes

Anthropogenic and natural phenomena can modify structural and functional traits of ecosystems leaving their imprint on the amount and seasonality of photosynthetic activity, an attribute of ecosystems that is strongly related with the Net Primary Productivity of the vegetation cover [1-3] and that often affects an array of processes ranging from biogeochemical cycling to human wellbeing. Species invasions, human migrations, changes in land use, disturbance regimes, atmospheric composition and deposition rates, and climatic conditions are some of the most prominent drivers of such functional changes [4-9]. In this paper we explore the ability of remote sensing data sets to assess functional ecosystem changes at the continental scale, focusing on the South American territory.

The strong functional changes that ecosystems have experienced in the last decades, and their derived consequences on society, demand a rapid detection, analysis, explanation and synthesis at a continental scale. This requires either large amounts of land-acquired information [e.g. 10] or statistical data, particularly for land cover changes detection [e.g. 11-13]. An alternative approach is based on the analysis of remotely-sensed data, which provides synoptic information over wide areas at temporal scales relevant to detect photosynthetic activity changes (both seasonal and inter-annual). This cost-effective approach may help scientists, policymakers and land managers to react timely and focus efforts on the most relevant environmental challenges and conflicts. Functional attributes of the ecosystem, like above ground primary productivity or evapotranspiration can be estimated through the combination of different bands of the electromagenetic spectrum [14]. The “Normalized Difference Vegetation Index” (NDVI), the most commonly used vegetation index, has being satisfactorily related to functional characteristics of vegetation, particularly with the fraction of photosynthetically active radiation intercepted by vegetation, and consequently with primary productivity [1-2, 15]. Long term series (∼20 years) of NDVI data, generated from coarse spatial resolution sensors, are valuable tools for the detection of both temporally discrete changes, like forest clearing, as well as gradual changes such as long term precipitation decline [16-17].

The “Advanced Very High Resolution Radiometer” (AVHRR) sensors, on board of the “National Oceanic and Atmospheric Administration” (NOAA) satellites, have provided one of the most extended time series of remotely-sensed data and continues producing daily information of surface and atmospheric conditions [18]. The original purpose of these missions, that began with the TIROS-N and NOAA-6 satellites in 1978, was to provide detailed information about atmospheric conditions of the world. But since a few germinal publications [c.f. 1, 19-21], and due to the continuous provision of information to present days, the AVHRR data has been used for many other purposes in geophysical, environmental or agricultural sciences [e.g. 22-24].

Satellite remote sensing data are subject to geometrical and radiometrical errors that may potentially reduce the utility of its information [25]. The main difficulties of the NOAA-AVHRR series stem from navigation uncertainties, sensor degradation and calibration, atmospheric contamination, orbital drift and limited spectral and directional sampling, among others [26-30]. Nevertheless, in several comprehensive efforts to improve data quality, different NDVI data series have been produced since the nineties following different processing streams [27, 31]. The most commonly used series were the “Pathfinder AVHRR Land” (PAL I and II versions) [32-33], the “Global Inventory Monitoring and Modelling Studies” (GIMMS) [26, 34], and the “Fourier-Adjustment, Solar zenith angle corrected, Interpolated Reconstructed” (FASIR) [35-36]. Because these series were originally developed for different purposes, they present several differences which generate uncertainties in the end users.

A critical merit of future AVHRR data sets, like the “Land Long Term Data Record” (LTDR, http://ltdr.nascom.nasa.gov/ltdr/ltdr.html), lies in their capacity to perceive real biophysical changes. For example, the sensitivity of the series to detect real changes in vegetation photosynthetic activity is central and needs testing; generating such empirical tests is a major challenge for the environmental science community. Moreover, corrections or improvements on the series are complex, and their details could be beyond the understanding and tracking possibilities of the general user-community (i.e. science, technology, and policy groups) [29]. These facts could cause in turn data up-dating and results' shearing difficulties.

### 1.2. Ecosystem changes in South America

In the last decades, almost all developing countries went through the fastest expansion and intensification of cultivation in their history [37]. In South America, besides climate alterations (natural or human induced) leading to changes in the productivity and phenology of natural vegetation [38-39], direct human drivers such as land use and land cover changes [40-43], infrastructure entreprises [44], and urban expansion [45-46] took place. Large extensions of native forests, savannas, grasslands and shrublands have been replaced by agriculture or tree plantations, mainly due to technological improvements and national and international new market conditions [47]. The dry forests of the continent experienced an important retraction, fragmentation and degradation [48-51], with a 12% area decline between 1980 and 2000 in the tropical zone [52].

The velocity and intensity of current ecosystem changes requires the development of efficient and integrated techniques of detection and quantification of the changes and their drivers, in order to project and assess future scenarios and impacts on the provision of ecosystem goods and services [53-54]. With its diversity of ecosystem changes, South America also provides an interesting arena to both evaluate the utility of the long-term satellite series (in particular the NOAA-AVHRR) and engage a flexible and multidisciplinary body of expertise supporting land use planning. Internet capabilities can bridge the gaps between disciplines and nations that prevail in the continent, engaging into a more informed discussion of ecosystem change assessment and management, even while scientific research is being developed [55].

In this paper we explore the possibilities and limitations of the three most commonly used NOAA-AVHRR NDVI temporal series (PAL, GIMMS and FASIR) to detect ecosystem functional changes in South America (associated or not with human intervention) and introduce a collaborative web-based utility to facilitate the use of these remote sensing tools. For these purposes we (1) compare spatial patterns of the trends of four NDVI attributes (annual average, annual maximum, annual minimum, and intra-annual coefficient of variation) among the three different long-term series; (2) analyze and compare the three NDVI data sets behaviour in five focus regions where ecosystems functional changes have been documented with independent data, and (3) introduce a collaborative web-based utility aimed to integrate ecosystem change knowledge across the continent based on the available long-term NDVI data sets.

## 2. Methods

### 2.1. NOAA-AVHRR NDVI data sets

The NDVI is computed as the ratio (NIR - R)/(NIR + R), where the NIR and R are the reflectance in the near infrared and the red portions of the electromagnetic spectrum detected by the sensors (0.72-1.1μm and 0.58-0.68μm in the NOAA-AVHRR, respectively). This spectral metric is strongly related with the fraction of the incoming photosynthetically active radiation intercepted by green vegetation [56-57] and it has been widely and satisfactorily used to describe the vegetation structure as well as its dynamics and functionality [58-59].

The NOAA-AVHRR NDVI data sets used here were: (1) the “Pathfinder AVHRR Land” (PAL, version I) [32], (2) the “Global Inventory Monitoring and Modelling Studies” (GIMMS) [26, 34], and (3) the “Fourier-Adjustment, Solar zenith angle corrected, Interpolated Reconstructed” (FASIR, version 4.13) [35, Los, personal communicaton, updated version of 36]. The series differ in their processing approaches (Table 1; for a genealogy of early NOAA-AVHRR data sets see Townshend [21]). The initial aim of PAL was to generate consistent long-term NDVI records based on physically adjustments (e.g. Rayleigh effect and Ozone corrections). For the same purpose GIMMS adopted a more empirical approach, avoiding full atmospheric correction and relying on an empirical-mode decomposition to remove outliers [27]. This series provided the most extended NDVI record (1981- 2006). Finally, FASIR was derived from PAL and developed as an input of Global Circulation Models. It included Fourier adjustment of outliers and a bidirectional reflectance distribution function that sought a common viewing and illumination geometry.

For the three series, original NOAA-AVHRR “Global Area Coverage” (GAC) data was discarded when solar zenith was greater than 80°, as the precision of the AVHRR visible channels degenerates rapidly in twilight areas [69]. In PAL this manipulation led to important problems, as winter data gaps were frequent in the NDVI series of high latitude areas in Southern South America. These gaps were exacerbated due to the frequent Patagonian winter-time cloud cover. Therefore, exclusively for PAL, we reconstructed the missing NDVI values of any given period (gaps were observed between April and August) averaging the available values for that period across the whole series. A failure in image provision by NOAA-11 generated another data gap between September 1994 and January 1995. We filled the missing data using a monthly-based average of the previous and following year. The NOAA-11 failure was solved in the GIMMS series using NOAA 9 data to fill this 4-month gap. In the FASIR series the problem was removed by an extrapolation of the NDVI record using a climatological mean and the Fourier Adjustment; particularly, two low-pass filters (±220 day and ±50 day moving windows, respectively) were used to interpolate the missing data [27, 34].

NDVI AVHRR data sets have been extensively used in the last two decades with different purposes, including land cover classification [71], vegetation structure/dynamics studies [72], fires assessment [73], energy balance calculations [74], among others. However, it is interesting to remark that there is large heterogeneity in the NDVI AVHRR data sets used for scientific purposes, being PAL, GIMMS and FASIR the most commonly used series. After a literature survey (Scopus database, http://www.scopus.com, March 2008), we quantified 81 papers using at least one these three data sets in the last 18 years (1989-2007), with PAL, GIMMS and FASIR being used in 55, 25, 8 publications, respectively. While PAL has been widely used since the 1990 decade, FASIR and principally GIMMS series have been increasingly used in the last five years.

### 2.2. NDVI trends calculation (1982-1999 period)

**Figure 1.**
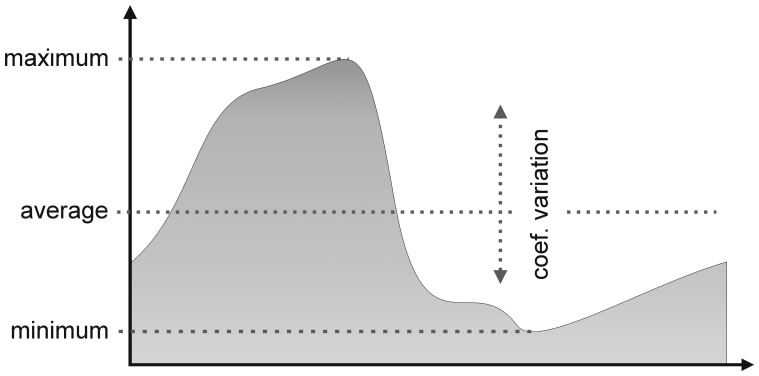
Scheme of the behaviour of the NDVI for a typical year of a deciduous forest, showing the variables selected to describe functional changes.

The temporal analysis of the changes in vegetation functionality was based in the NDVI spectral metric. We selected four variables to characterize possible functional changes in the amount and seasonality of the photosynthetic activity: average annual NDVI, maximum annual NDVI, minimum annual NDVI and NDVI intra-annual coefficient of variation (Figure 1). Average annual NDVI has been used as a surrogate of annual primary productivity [15, 75-76], and thus is sensitive to changes between systems with different productivity. Minimum values are strongly sensitive to the replacement of natural vegetation, particularly evergreen covers by cultivation [77]. Maximum values are sensitive detecting land use changes in resources-limited areas, like the irrigation in semiarid regions or the use fertilizers in poor soils areas. The NDVI intra-annual coefficient of variation captures the seasonality of the vegetation, representing thus the variation of the primary productivity through the year. Raising winter temperatures in temperate or cold areas or the replacement of deciduous by evergreen species could cause declines in this variable. The study period selected was 1982-1999, in where the three NDVI series overlap. To reduce the possible effects of low quality NDVI values (off-nadir values and pixels with water vapour contamination) on trend calculation [69], we used monthly composites generated by the maximum value composition technique [60].

In order to analyze the 18-years trends or temporal anomalies for each data set and each NDVI variable, we performed a pixel-based linear regression. We assumed that pixels experienced functional changes (positive or negative) when the slope of a given NDVI variable vs. time relationship was significantly different from zero (H_o_: β=0, H': β≠0; p<0.1). Despite possible statistical limitations [78-79], this technique was selected for its simplicity and because has been successfully applied in other works [30, 70, 80-83].

Finally, for the three NDVI series we estimated the number of pixels of the three possible trend classes (positive, negative, and no change) and analyzed the convergences and divergences of the trend results among the series. Based on overlapping maps of the results, we generated three square matrixes counting the number of pixels that fitted six possible results for a given pair of series: (1) no change/no change, (2) positive/positive, (3) negative/negative, (4) positive/negative, (5) positive/no change, and (6) negative/no change. We performed these calculations exclusively for trends on average annual NDVI.

### 2.3. Analysis of spatial pattern of changes

The set of maps of functional changes generated by PAL, GIMMS, and FASIR may differ, not only on the number of pixels displaying significant trends, but also in the spatial aggregation of changes. We explored the degree of aggregation of pixels with negative or positive trends using the O-ring statistic [84, 85]. This function is related to the standard variogram techniques and to the Ripley's K-function, commonly used in geostatistics [e.g. 86]. But, instead of using concentric circles to compute the density around the target point as the Ripley's K-function, the O-ring statistic used rings or bands of certain width and at different distances *r* to the target point. Therefore, the spatial pattern at fine scales does not directly influence the spatial pattern at coarser scale because these points are not contained in the ring. The function *O(r)* is the expected number of points within a ring with radius *r* from an arbitrary point. If points are randomly distributed in space (Poisson distribution), the expected result of *O(r)* equals the overall density of points. Spatial pattern showed in three series of images were compared with the spatial pattern expected under the null model of Completed Spatial Randomness (CSR) by generating confidence bands using Monte Carlo simulations (n=100, α=0.05). Values of the O-ring statistic higher than the ones predicted under the null model indicate aggregation, while smaller values indicate repulsion [for more details, see 84]. In all cases, density of changes in each set of images were preserved and only univariate patterns were explored (i.e., aggregation of positive or negative changes within themselves). We worked with a subset of ∼9,250,000 km^2^ (∼52 % of the image) in which water pixels were masked. Computations and graphs were done using “Programita” software, version 2004 [c.f. 85]. We only explored the patterns of average annual NDVI trends.

### 2.4. Independent evaluations in changing regions

**Figure 2.**
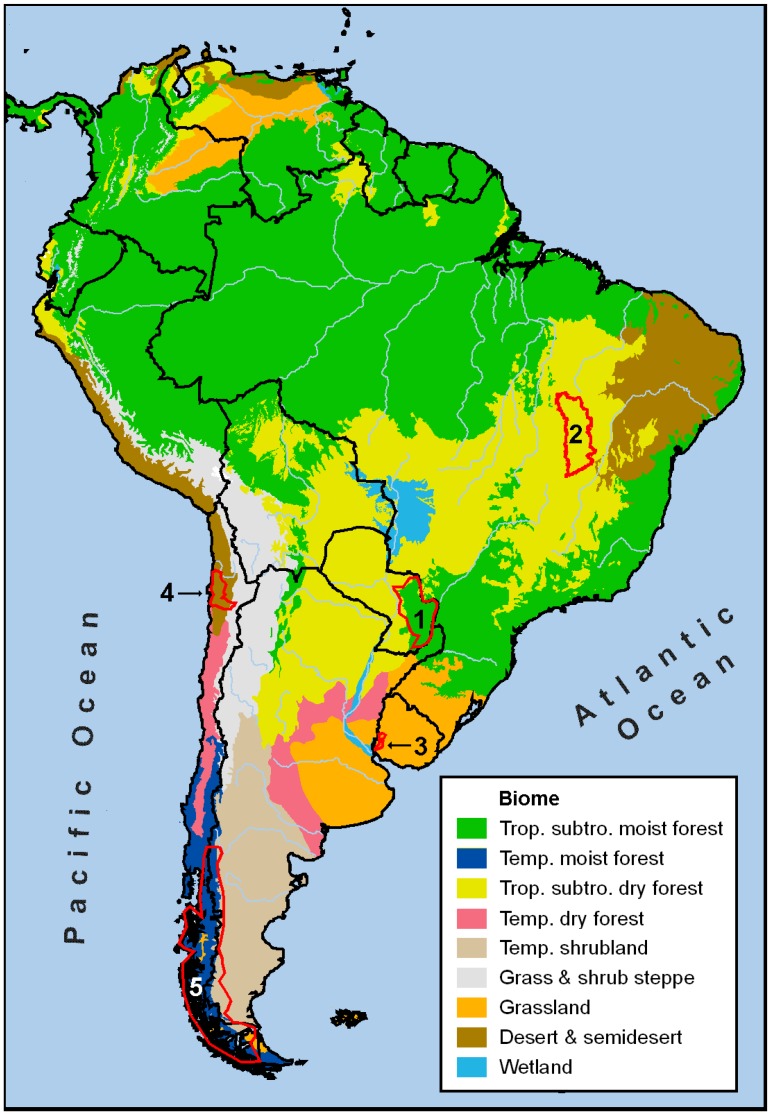
Localization of the five focal regions and major biomes in South America. The focal regions are: (1) Eastern Paraguay, (2) Western Bahia –Brazil, (3) Uruguay River margins – Argentina, Uruguay, (4) Northern Chilean deserts, and (5) Patagonian Andes – Argentina, Chile.

In order to evaluate the observed NDVI trends, we explored and described five focal regions involving positive and negative changes (Figure 2, Table 2). For each one, we collected enough evidences from different sources to assume a direct or indirect causality among the changes in vegetation productivity and environmental (land use/cover or climate) changes.

## 3. Results and interpretations

### 3.1. NDVI trends calculation (1982-1999 period)

**Figure 3.**
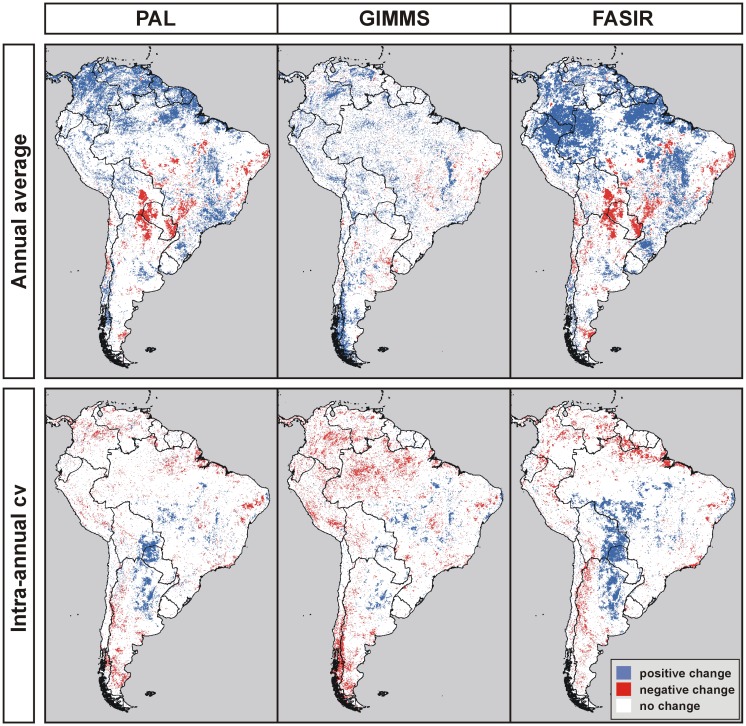

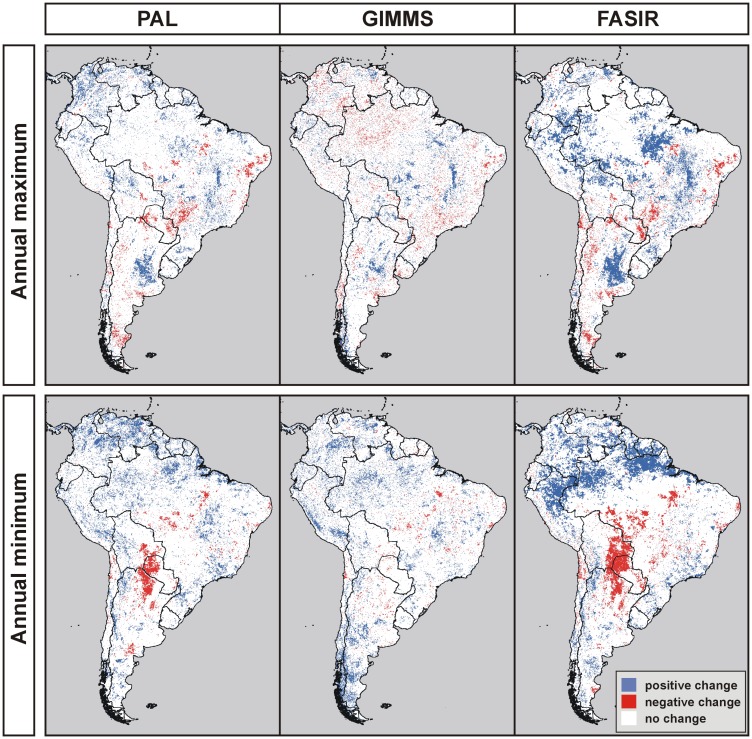
Trends of average annual NDVI, maximum annual NDVI, minimum annual NDVI and intra-annual coefficient of variation of NDVI in South America (H_o_: β=0, H': β≠0; p<0.1), for the FASIR, GIMMS and PAL series (1982-1999 period).

Several differences arose in terms of the patterns of change, i.e. the total number pixels with significant changes, their localization, and their sign for the four variables and three series (Figure 3). The total number of pixels displaying NDVI significant changes differed across the series, with FASIR presenting the highest proportion (32.7%) and GIMMS the lowest (16.2%, Table 3a). PAL and FASIR data sets were more consistent among them than GIMMS, with a convergence of the results in 71.2% of the ∼285,000 land pixels, while GIMMS and FASIR displayed important divergences, with a 37.8% of the pixels showing different trends (Table 3b). The similarity between PAL and GIMMS was mainly related to the large number of pixels with no changes in both series (65.2%). These results contradict the earlier suggestion that FASIR and GIMMS should have a better match of interannual anomalies than their corresponding match with PAL since they were originally designed to provide top-of-the atmosphere reflectance values [27, 29] (Table 1). The fact that FASIR series were compiled using PA may have strongly influenced the trends.

### 3.2. Analysis of spatial pattern of changes

**Figure 4.**
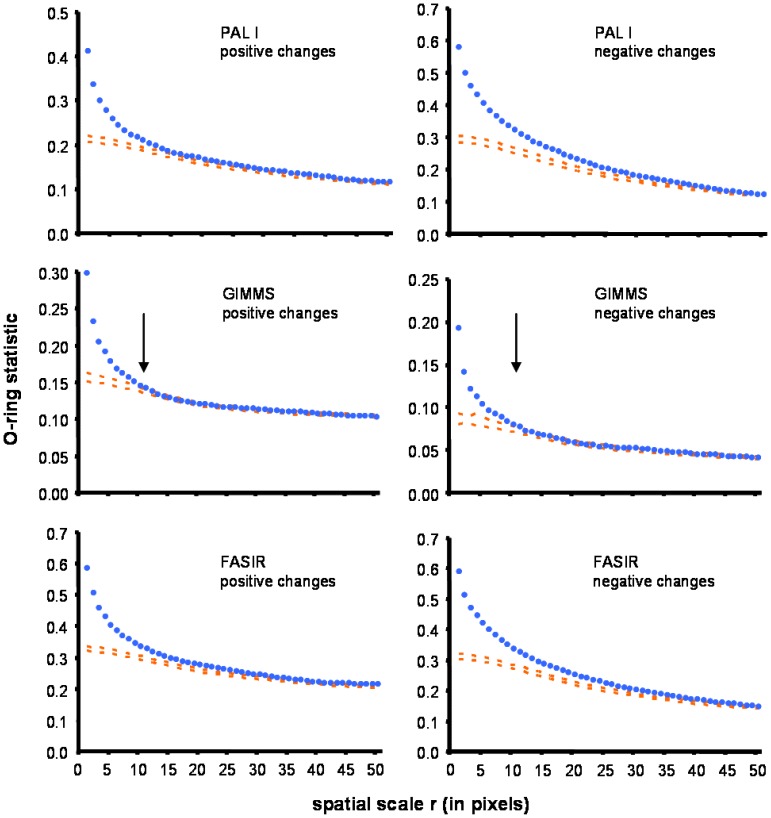
Spatial association of positive and negative changes showed in FASIR, GIMMS, and PAL series based on the O-ring function. Blue dots indicate the observed pattern and orange dashed lines corresponded to 95% confidence bands for a null random model generated by Monte Carlo simulations. Observed values above the orange indicate aggregation. Arrows indicate the scale at which observed patterns converge with the null model (FASIR and PAL arrows are beyond the shown x scale).

Even though positive and negative changes showed substantial aggregation in the maps generated for the three sets of images, differences in aggregation scale emerged among them (Figure 4). The degree of aggregation was higher in FASIR and PAL series (>50 pixels or 400 km) than in GIMMS, which showed aggregation only at fine spatial scale (≤11 pixels or 88 km). These patterns indicate differences in the size of the patches showed in the maps generated by PAL and FASIR, with respect to GIMMS series. Although big patches were visible in all three series, their spatial cohesion was better defined in FASIR and PAL series (Figure 3). Since the effects of ecosystem changes drivers on the NDVI (such as deforestation or rising temperatures) have an aggregated spatial expression at a regional scale, our results shows for this analysis a larger reliability for the last two series.

### 3.3. Independent evaluations in changing regions

The convergence and divergence analyses and the evaluation with independent information for the five focal regions (Table 4), suggested higher accuracy in the detection of ecosystem changes in PAL and FASIR series than in GIMMS, as they reflected the changes analyzed with independent data in a larger number of pixels and spatially more aggregated (see the regions particular results).

#### 3.3.1. Eastern Paraguay

This region experienced rapid forest to agriculture conversion throughout the last three decades [91], with native forest cover occupying 70, 40, and 25 of the area in 1973, 1989, and 2000, respectively [42]. The deforestation rate approached 150,000 ha year^-1^ in the 1973-2000 period [42]. The development of infrastructure and the introduction of the soybean crop in the 1970s was a key driver on this deforestation process [92]. In the last 15 years, the soybean area increased dramatically from 530,000 ha in 1991 to 1,800,000 ha in 2005 [93]. Currently, the frontier of soybean cultivation moves westward because most of the Alto Paraná Atlantic Forest has been already deforested, covering only about 7% of the entire region area [93].

This extensive and deep land use change would lead to a decrease of average annual NDVI because of the significantly lower productivity values of rain-fed croplands compared to broadleaf evergreen forests [94]. Lower minimum NDVI and higher seasonality (higher intra-annual coefficient of variation) would also be expected outcomes of this land use change, owing to the inclusion of a fallow period in agricultural rotations, during which vegetation is not allow to growth in the field. Supporting these predictions, a decline in the annual and minimum NDVI was clearly evidenced in PAL and FASIR series but surprisingly not in GIMMS (Table 4 and Figure 3), albeit this data set is believed to have important improvements over the PAL series [95]. An increase of the NDVI intra-annual coefficient of variation was also evidenced in the three NDVI data sets.

This area presents sharp contrasts across national boundaries, offering further evaluation for the observed NDVI trends. While in eastern Paraguay an intense deforestation and agricultural expansion took place during the study period, in the adjacent countries (Brazil and Argentina) no significant land use change occurred in the same time span. In the Brazilian territory agricultural expansion took place in the first half of the 20^th^ century and the crop area remained high and stable throughout the study period. By contrast, in the adjacent Argentine territory, native forest has been protected and little conversion to agriculture occurred. These different trajectories of land use changes would have brought about ecosystem functioning changes in the Paraguayan territory, but not in Brazil or Argentina. In concordance with this analysis, significant trends in NDVI variables were only observed in eastern Paraguay and no clear change was evidenced in the adjacent countries (PAL and FASIR; Figure 3).

Finally, as a finer example in this focal region, the Yacyretá hydroelectric dam started to be constructed at the end of 1983, replacing the original forest with a 1,600 km^2^ water body in 1994. Surprisingly, for the four variables, PAL and FASIR represented this significant change, while GIMMS almost completely eliminated the signal.

#### 3.3.2. Western Bahia –BR

The western counties of the Brazilian state of Bahia were covered by dry forests, woodlands and savannas until the beginnings of the 1980 decade, when a massive advance of agriculture activities took place (Table 2). As we could not find quantitative analyses of this land use change, we examined the area under agriculture following two complementary sources of information: 1) 1982-1999 remote sensed data, and 2) 1990-1999 county-level agricultural statistics of the “Produção Agrícola Municipal” [96]. For the first approach, we extracted and corregistrated Landsat MSS and TM quickviews from the INPE webpage (Instituto Nacional de Pesquisas Espaciais, http://www.inpe.br/index.php), one for each year (scenes WRS1 236/068-069 and 237/068 and WRS2 220/068-069).

**Figure 5.**
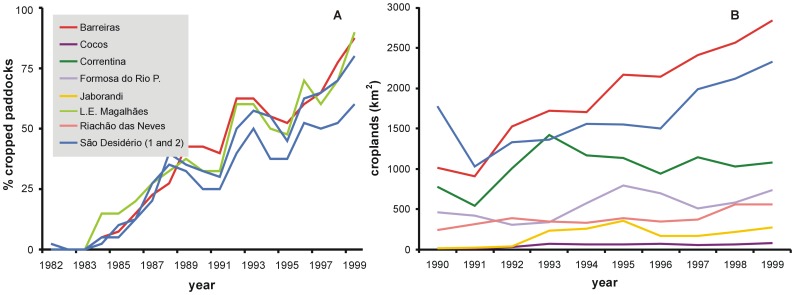
**(a)** Percentage of the area under agricultural activities measured through remote sensed information (1982-1999 period) for the three main counties (Barreiras, L.E. Magalhães and São Desidério), and **(b)** area in square kilometres under agriculture from statistics (1990-1999 period) for the entire region [96].

We established four random transects over the current agricultural areas of the counties of Barreiras, L.E. Magalhães and São Desidério (that comprise the core area of the region) of 600 km^2^ divided in ten subunits, and in each subunit we visually estimated the plots percentage of the subunit (five potential values, 0%, 25%, 50%, 75 and 100%), and summarized the information for the whole transect for each year. For the second approach we extracted for the eight western counties of Bahia the yearly-based total sown area (with the exception of the county L.E. Magalhães, as data was missing). We found in both approaches a steady increase in cropped area since the beginnings of the 1980' decade, from almost no cropped area to a cultural landscape (Figure 5). This increase was highly associated with -for example- the mean FASIR NDVI behaviour for the first and the second data sources (r^2^=0.59 and 0.44, α=0.05, respectively).

This cropland expansion was highly associated with improvements in soil management, technological investments and the application in 1984 of irrigation programmes. These enhancements allowed the development of a wide diversity of cultures (cotton, coffee, soybean, bean, maize) [97]. According to IBGE statistics, in the state of Bahia the irrigated areas went from 700 km^2^ in 1980 to 2,100 km^2^ in 1996. In the analyzed counties, the percentage of area equipped for irrigation in 2000 [98-99] was in average 1.25%, but reached up to 35% in some of the pixels of 5 minutes of spatial resolution. Moreover, with the aid of the Google Earth and only in the São Desidério county, over 160 on-use central pivot rain fed plots were found, with an average area of 950 ha, constituting a 1.5% of the county's area. This value would constitute the minimum irrigated value of the region, as square-shaped irrigated plots also exist.

By contrast with Eastern Paraguay, in this region, with high evapotranspirative demands and oligotrophic soils (Table 2), the irrigation and the building-up soil fertility [100] would imply a raise in vegetation productivity compared with natural ecosystems. The implications of this land use change were evidenced by increases of average, maximum and NDVI coefficient of variation of the three series (Table 4). Even though the area occupied by the irrigated plots was small, their NDVI signals may have been amplified by the spatial and temporal maximum value composite techniques (Table 1).

#### 3.3.3. Uruguay River margins – AR, UY

This small region has experienced a very fast expansion of tree plantations (mainly eucalyptus and pines) over native grasslands since the beginning of the 1990 decade that continues to the present [43]. This process was motivated by beneficial governmental policies together with suitable environmental and market conditions [101]. In Uruguay, the afforested area increased from 180,000 has in 1980 to 660,000 in 2000 [102]. Particularly, in the Uruguay River margin (Paisandú and Rio Negro counties) the afforestation rate increased almost an order of magnitude (from ∼850 ha year^-1^ in 1980 to ∼7,500 ha year^-1^ in 1999) [103]. Argentina follows a similar trend, being the annual afforestation rate quintupled in the 1992-2000 period (from 20,000 to 100,000 ha year^-1^) [104]. In the Argentinean margin of the Uruguay River (Concordia and Colón counties) the afforested area was almost doubled between 1988 and 2002 (39,000 and 73,000 ha year^-1^, respectively; Censo Nacional Agropecuario).

This deep land use transformation led to profound ecosystem functional changes. Aboveground measurements of net primary productivity (ANPP) made in the region suggest that eucalyptus plantations tripled the ANPP of native grasslands [105-108]. Afforestations also decreased the seasonal variation of primary productivity, as suggested by the fraction of PAR intercepted (FPAR) by canopies through the year [14].

The increased productivity of tree plantations was confirmed by a significant positive trend of the NDVI annual average detected by PAL and FASIR data sets, but not by GIMMS (Table 4). This trend was mainly explained by sharp increase of the NDVI annual minimum and secondarily, by a lower increase of the NDVI annual maximum (Table 4). A decline of the intra-annual NDVI coefficient of variation was also evidenced by PAL and FASIR data sets.

#### 3.3.4. Northern Chilean deserts

This virtually uninhabited territory is one of the driest places in Earth, leading to an almost barren landscape devoid of plant life (Table 2). Isolated vegetation formations can only develop in river courses, in marine fog-fed gorges and coastal slopes, and in basins where occasional water accumulation occurs [109-111]. As a desert ecosystem, the pulses of rainfall result in increased soil moisture levels that regulate the pattern of biota productivity [112-113], particularly in lowlands systems [114]. The region is –with the exception of cooper mines- unexploited, and no significant land use change have undergone during the study period, contrasting with the three previous analyzed areas. But, since the second half of the 19^th^ century, a trend of decreasing precipitation have been detected for Central and Northern Chile [115-116]. This drying trend is not related with recent increase in ENSO intensity, frequency and duration that indeed have helped to alleviate the drought in recent decades [116]. The degree to which El Niño - Southern Oscillation (ENSO) phenomenon determines or control the climatic conditions (precipitation, temperature, coastal fog) in the northern area of the country is spatially and seasonally complex and is still an issue of debate [116, 117].

**Figure 6.**
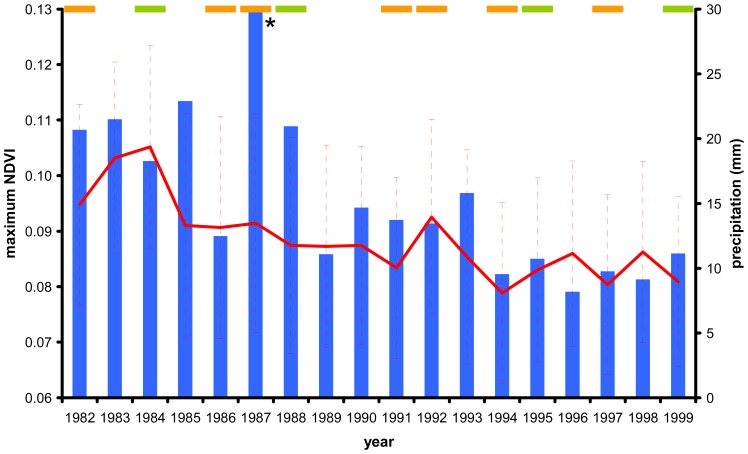
Northern Chilean deserts FASIR maximum annual NDVI during the 1982-1999 period for 222 significan negative 8×8km pixels (red lines indicates the trend and vertical bars the standard dev tions), annual accumulated precipitation (blue bars) for the approximate region delim ted by the -22.25° to -24.25° latitude and -69.25° to -70.75° longitude from the CR TS 2.1 [118], and El Niño or La Niña years (orange and green upper horizontal bars, respectively), according to the “Multivariate ENSO Index” (MEI) [119] and “El iño & La Niña Years: A Consensus List” (http://ggweather.com/enso/yers.htm). The correlation coefficient (r^2^) between MEI and NDVI annual maximum values was 0.03 (α=0.05). * for the year 1987 the precipitation was 86 mm.

Even though the three NOAA-AVHRR NDVI series have been corrected for sensor degradation using invariant targets of extreme deserts [28, 61] (Table 1), we surprisingly found in this region -for the average, minimum and maximum variables- significant and negative NDVI trends for the three series (Table 4). These decays were associated with the mentioned historical precipitation decay, finding -for example- a high association of this climatic variable with the maximum FASIR NDVI behaviour (r^2^=0.44, α=0.05, excluding 1987 year; Figure 6). As stressed before, the application of maximum value composite techniques, could have amplified the NDVI signal of the very small and isolated vegetated areas (Table 1). Finally, 350 km to the Northeast of the Northern Chilean deserts, the Uyuni salt plains -an area free of vegetation in South America- presented no NDVI changes for all the variables and the PAL and GIMMS series (in FASIR, the area is masked), possibly supporting that the changes in the first area would not be caused by computational or sensor artefacts.

The pattern of decreasing NDVI continued southward up to the Mediterranean Matorral, a region that was also the scenario of one of the most severe precipitation reductions since the second half of the 19^th^ century [115-116, 120].

#### 3.3.5. Patagonian Andes –AR, CL

The forest-dominated Patagonian Andes, as well as most of southern South America, experienced during the last century a general positive trend in the mean annual temperature, intensified in the last three decades [121, 122]. The warming rates approached 2-3 °C per century in the region and it is mostly explained by an increase in minimum temperatures [121]. Since in this area the growth of native forests is mainly constrained by temperature [123-125], this climatic change has led to a increase in vegetation growth, clearly evidenced since the 1970 decade [123, 126].

The increased vegetation growth previously observed by tree-ring analysis was detected in the three NDVI data sets through a positive trend of the NDVI annual average, especially in the GIMMS series (Table 4). This pattern is mostly explained by an increase of NDVI minimum values (Table 4), which is supported by the raise in minimum temperatures detected in long term climatic data [121]. As a consequence of this NDVI minimum increase, a decline of NDVI intra-annual coefficient of variation was also observed in PAL and FASIR series (Table 4). These increasing trends of the NDVI match observations in high latitudes of the northern hemisphere where, increasing NDVI values were highly associated with higher temperatures [70, 127].

## 4. General discussion and concluding remarks

Comparison of areas of known functional ecosystem changes with observed remotely sensed patterns, together with spatial aggregation analysis (O-ring function), and cross-series correlations, allowed us to explore the ability of the three NOAA-AVHRR NDVI series to detect patterns of change in the South American continent. The FASIR and PAL series identified functional shifts or steady changes clearly associated with land cover changes or with climate conditions (precipitation, temperature) in very different environments. We also found -at least for South America- that the FASIR and PAL data sets were both more consistent and more accurate than the GIMMS dataset, a fact that remained unknown before our analyses [29].

Surprisingly, beyond the many divergences emerged among the three series, we found that GIMMS data have almost eliminated some of the most prominent patterns of ecosystem change over the continent, as shown for the Eastern Paraguay, Uruguay R. margins and Central Chile focal regions (Table 3). Our results -based on the spatial patterns rather than in the NDVI values- contradict the previous notion that GIMMS vs. FASIR had greater similarities among them than compared to PAL because they provide reflectance values at top of the atmosphere [29]. Although GIMMS has been proposed as a more accurate tool in comparison with PAL due to a higher correlation with the newest (and thus more accurate) SPOT-4 VGT data [128], we have shown that it has a poorer ability to detect long-term changes, possibly the most valuable application of this type of satellite data.

Two major challenges arise in the detection of current or past terrestrial functional changes through remotely sensed data sets. The first one is related to the assignation of a change signal to surface biophysical phenomena, when the driving cause is a measurement or processing artefact (equivalent to commit a type I error). The second is associated with the removal of real variation during the data acquisition and processing stages. This may be considered a type II error and could be very important in multi-instrument, multi-date satellite data like NOAA-AVHRR NDVI series [34]. In both points, the reliability of the results (as a representation of reality) should be accomplished in the process of evaluation with independent field observations, theoretical models, and other remotely sensed derived information. In particular, field observations could involve structural or functional ground truth data at a landscape to regional scales (carbon stocks, leaf area index, phenology, biomass yields) [24, 129, 130], or information on shifting conditions for variables such as temperature or precipitation in ecosystems in which they are well known drivers of primary productivity [131].

As stressed in this study, different sources of remote sensed data might lead to different results. The technical explanation of these differences remains unclear and the exploration would require specific analyses of the consequences of the different manipulations or treatments that the original information underwent. Further research, like cross-calibration with new satellite sensors (MODIS TERRA or AQUA), and long-term ground measurements of structural and functional data (like eddy covariance carbon flux measurements) are needed in order to enhance the quality of future long term satellite series [24].

Academia or research communities – like any other social group – adopt distinctive specific behaviours, namely the (mostly implicit) acceptance of guidelines in relation to what information and methodologies ought to be used or implemented. These guidelines undoubtedly drive the knowledge development process, widening the perspective of research and enhancing its quality, but these research standards could have a deleterious impact if they are not updated in the light of new theoretical or empirical opportunities. Restrictions in the use of alternative sources of information (like the now unavailable PAL series) could lead to biases in the predictions and in the areas of research emphasis (this might be the case if -for instance- GIMMS would be the only accepted series). In addition, departure from these guidelines could lead to a worthless effort in relation to time, money, human and computational capability, as the information generated following them could be not accepted by the general community. One of the main findings of this study is that the conclusions (particularly about ecosystem functional changes), should not only be evaluated at the level of assumptions (i.e. physical or mathematical aspects of image processing), but also at the level of results, without being this last evaluation a redundancy or a circular argument.

### The LechuSA initiative

The “Land ecosystem change utility for South America” initiative (http://lechusa.unsl.edu.ar) is an online collaborative space that seeks to identify and comprehend the most important functional changes of the terrestrial ecosystems of South America. To achieve this objective, we encourage the participation of local experts able to recognize the nature and causes of these changes.

**Figure 7.**
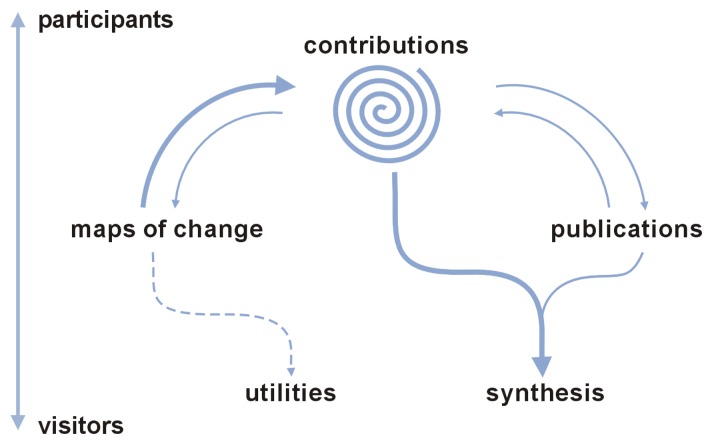
Schematic representation of the different components of the LechuSA initiative, indicating the flows of information and the different compromise of the scientific community (from visitors to participants of the forums).

Analyzing satellite images, in particular of NOAA-AVHRR NDVI temporal series, we have identified “hotspots” that are likely to experience intense functional change (“maps of change” in Figure 7). We consider that these maps could be the basis for general ecosystemic discussions and particular testing of long term NDVI capabilities and problems (“contributions” in Figure 7). Thus, in order to guide the experts in the contribution of the possible causes of these changes, we ask them to: 1- explore the generated maps, 2- select a “hotspot” of change, 3- suggest the possible causes of the detected changes, by the formulation of hypotheses or presentation of evidence with different degree of support, and 4- present their related publications about the discussed changes (“publications” in Figure 7). Another goal of the initiative is to facilitate access to satellite imagery databases to a broad nonexpert public. This will in turns bridge the gap between field scientists (focused on local scale patterns), and remote sensing experts (focused on continental to global scale patterns) (“utilities” in Figure 7). All the information is accessible for the general public without any kind of restrictions and absolutely free. The different participants would be able to perform their own meta-analyses or synthesis and publish them with a proper citation of the sources of information (“synthesis” in Figure 7).

## Figures and Tables

**Table 1.** Description of the three NDVI NOAA-AVHRR data sets used in this study [adapted from 27, 30, 31, 34, Los personal communication, updated version of 36, 64, 69, 70].

	***PAL***	***GIMMS***	***FASIR***
*Data Set Origins (and its spatial resolution)*	NOAA-AVHRR GAC 1B (4 km)	NOAA-AVHRR GAC 1B (4 km)	Radiance in PAL dataset (8 km) [32]
*Instrument and change in times*	7, 9, 11, 14	7, 9, 11, 9 (descending), 14, 16	7, 9, 11, 14
*Known temporal span*	1981-2001	1981-2006	1982-1999
*Temporal resolution*	10 days	15 days	10 days
*Spatial resolution*	8 km	8 km	8 km
*Spatial compositing*	Forward, nearest neighbor mapping. Selection of the 4 km pixel with the maximum NDVI value for the 8 km output bin. Only pixels within 42° of nadir are considered.	Forward, nearest neighbor mapping. Selection of the 4 km pixel with the maximum NDVI value for the 8 km output bin.	Inherited from PAL series.
*Temporal compositing*	Maximum NDVI values composition of the 10-days images [60].	Maximum NDVI values composition of the 15-days images [60].	Inherited from PAL series.
*Radiometric corrections*	Calibration with pre-flight constants modified by degradation over time [61], based on the use of desert invariant calibration targets.	NOAA-7 to NOAA-14 channels 1 and 2 calibrations using the Vermote and Kaufman parameters [62]. NOAA-16 channels calibrations using pre-flight values. Data further adjustment using invariant desert targets [28].	Data correction following the Los technique of invariant desert targets [28].
*Viewing and illumination corrections*	No specific corrections have been applied.	Correction of illumination and viewing angle effects using the adaptive empirical mode decomposition (EMD) method [63].	Correction of illumination and viewing angle effects with Bidirectional Reflectance Distribution Function (BRDF) techniques for Pathfinder radiances [64].
*Cloud corrections*	Based on Cloud Advanced Very High Resolution Radiometer (CLAVR) algorithm [65].	Based on thermal band.	Based on thermal band and reconstruction of tropical evergreen broadleaf vegetation data with a maximum filter.
*Stratospheric aerosols correction*	No corrections.	Volcanic aerosol correction for 1982-1984 and 1991- 1994 [66, 67].	Volcanic aerosol correction for 1982-1984 and 1991-1994 [66].
*Molecular absorption and scattering corrections*	Ozone absorption from the Total Ozone Mapping Spectrometer (TOMS) data set, and Rayleigh scattering [68].	No corrections.	Inherited from PAL series.
*Manual checking*	On every layer of the composite files.	On navigation accuracy, data drop outs, bad scan lines, and other strange values.	On navigation accuracy, data drop outs, bad scan lines, and other strange values.
*Noise attenuation*	Not applied.	Removal of noise and attenuation of cloud and missing pixels effects with Kriging interpolation.	Replacement of extreme outliers and missing data with the long-term mean. Posterior restoration of outliers caused by cloud interferences and short-term atmospheric effects through Fourier Adjustment.
*Scaling procedures*	Not applied.	Match with SPOT Vegetation NDVI data during overlapping period [34].	Data extrapolation for winter needleleaf evergreen areas.

**Table 2.** Main physical characteristics of the focal regions used to evaluate NDVI trends results. Acronyms: MAP, Mean annual precipitation; MAT, Mean annual temperature; MAPET, Mean annual potential evapotranspiration; AR, Argentina; BR, Brazil, CL: Chile; UY, Uruguay. Ecoregion name following Olson et al. [87], climatic information was extracted from CRU 2.0 [88] and soil type and land form from the SOTER-Latin America and the Caribbean [89] databases. The potential evapotranspiration was calculated using the same database and following Penman-Monteith method [90]. The calculation of the areas affected by changes was extracted from the superposition of the three series' average NDVI.

***Region***	***Eastern Paraguay***	***Western Bahia –BR***	***Uruguay River margins – AR, UY***	***Northern Chilean deserts***	***Patagonian Andes –AR, CL***
*Area affected (km^2^)*	83,300	65,700	3,400	24,400	370,000
*MAP (mm)*	1500-1800	1050-1750	1100	0-50	700-4500
*MAT (°C)*	21.0-23.5	23.0-24.5	18.0	13.0-15.0	5.0-7.0
*MAPET (mm)*	1200-1300	1400-1450	1150	1150-1350	500-850
*Soils*	Acrisol, Arenosol	Ferralsol	Phaeozem, Vertisol	Regosol, Solonchak, Leptosol	Andosol, Cambisol, Leptosol, Phaeozem
*Land forms*	Plains to medium- gradient hills	Plateaus	Plains	Depressions to medium-gradient hills	Medium to high gradient hills and mountains
*Ecoregion (and vegetation type)*	Alto Paraná Atlantic (moist) Forest	Cerrado woodlands and savannas	Humid Pampa (prairies and grass steppes) and Uruguayan Savanna	Atacama Desert	Magellanic subpolar and Valdivian Temperate forests
*Land use*	Diversified dryland agriculture and grazing	Diversified agriculture with irrigation	Tree plantations and grazing	Negligible	Conservation, grazing and wood extraction

**Table 3.** In percentage of the total number of pixels, **(a)** distribution of the pixels in each of the three trend classes, and **(b)** convergences and divergences for the trend results among the three NOAA AVHRR data sets for the average annual NDVI. Acronyms: ++ positive/positive, -- negative/negative, *00* no change/no change, +- positive/negative, +*0* positive/no change, and -*0* negative/no change.

***(a)***	***PAL***	***GIMMS***	***FASIR***
*Positive changes*	19.3	13.3	26.7
*Negative changes*	5.0	2.9	6.0
*No changes*	78.4	86.6	67.3

***(b)***	***Convergences***				***Divergences***			
	**++**	**--**	***00***	***total***	***+*-**	***0+***	***0*-**	***Total***

*PAL vs. GIMMS*	3.4	0.3	65.2	68.9	0.8	24.2	6.3	31.3
*PAL vs. FASIR*	10.9	2.8	57.5	71.2	0.1	23.5	5.2	28.8
*GIMMS vs. FASIR*	4.4	0.3	57.5	62.2	1.1	29.6	7.1	37.8

**Table 4.** Synthesis of the trends for the four NDVI variables in the five focal regions used to evaluate the results. The signs indicate the direction of changes and the approximate area affected by the change (more signs, larger area in the focal region). Acronym: cv, coefficient of variation.

***Region***	***Annual average***			***Annual maximum***			***Annual minimum***			***Intra-annual cv***		
	***PAL***	***GIMMS***	***FASIR***	***PAL***	***GIMMS***	***FASIR***	***PAL***	***GIMMS***	***FASIR***	***PAL***	***GIMMS***	***FASIR***
*Eastern Paraguay*	- - -	0	- - -	- -	+	- -	-	0	- -	+	+	+
*Western Bahia*	++	++	+++	++	++	+++	0	0	0	+	++	+
*Uruguay R. margins*	+++	0	+++	+	0	+	++	0	+++	-	0	- - -
*Northern Chilean deserts*	- -	-	- - -	-	0	- - -	- -	- -	- - -	0	0	0
*Patagonian Andes*	+	+++	++	0	++	0	+	+++	+	- -	- - -	0
